# One-pot synthesis of thermally reversible materials using maleimide-polysaccharide and furan-lignin derivatives

**DOI:** 10.1039/d5ra02344k

**Published:** 2025-08-08

**Authors:** Valentin Silveira, Raffaello Papadakis, Stergios Adamopoulos

**Affiliations:** a Department of Forest Biomaterials and Technology, Swedish University of Agricultural Sciences Vallvägen 9C 756 51 Uppsala Sweden stergios.adamopoulos@slu.se +46 73-572 27 86

## Abstract

The bio-based materials potato starch (St) and Kraft lignin (KL) were chemically modified to create a thermally responsive network through a reversible Diels–Alder (DA) reaction between maleimide and furan groups present in St and KL, respectively. To achieve this, St was esterified in a one-pot synthesis at room temperature with 6-maleimidohexanoic acid (6-MHA) to produce St 6MHA aligning with the 12 principles of green chemistry, which was confirmed by FTIR, ^1^H, ^13^C, and 2D NMR spectroscopy. Furan (Fu) groups were introduced to KL by reacting furfuryl glycidyl ether with the phenol entities of KL, forming KL-Fu. The structures of the KL-Fu derivatives were characterized using FTIR, ^1^H, ^13^C, and ^31^P spectroscopy, as well as TGA. St 6-MHA and KL-Fu were then subjected to thermal cycloaddition through the DA reaction. Furthermore, controlled retro-DA reactions were induced thermally and confirmed by FTIR and ^1^H NMR spectroscopy. DSC analysis of the final products revealed the thermally responsive nature of the system. This study highlights the significant potential of such a thermally responsive system, demonstrating that effective chemical modification of abundant renewable feedstock can enable the development of high-value materials thereof.

## Abstract 2

The bio-based materials potato starch (St) and Kraft lignin (KL) were chemically modified to create a thermally responsive network through a reversible Diels–Alder (DA) reaction between maleimide and furan groups grafted on St and KL, respectively.

## Graphical abstract

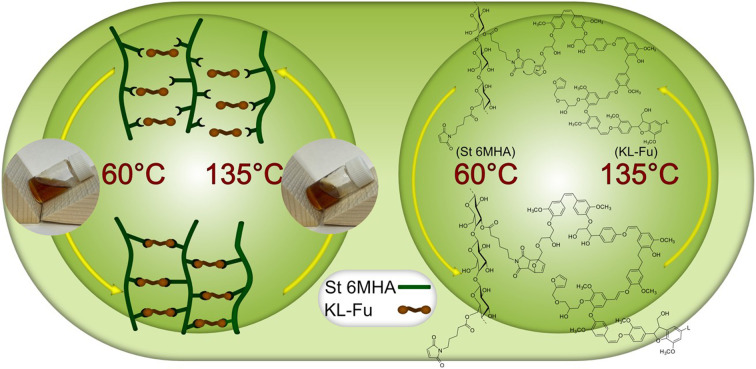

## 1 Introduction

The persistent use of fossil fuel-based materials has led to significant environmental challenges, including pollution and resource depletion. As a sustainable alternative, biopolymers have emerged as promising solutions due to their biodegradability and renewable nature. Among these, starch and lignin, derived from plant biomass, have gained attention for their potential applications.^1,2^ However, these biopolymers often exhibit poor performance when used in material applications in terms of mechanical properties, thermal stability, water resistance and others, limiting their broader use.^3,4^ When used alone, these biopolymers lack sufficient mechanical strength or water resistance to function effectively as standalone materials. Consequently, they have primarily been utilized as fillers in traditional materials for enhancement of their bio-based content or similar.^5^ To address these limitations, researchers have explored various modification techniques. For starch, esterification^6^ and oxidation have traditionally been employed to enhance water resistance^7^ and facilitate crosslinking. Meanwhile, research on lignin has primarily focused on alkylation, oxidation, and polymerization for applications as coatings, adhesives, and emulsifiers.^8,9^ The growing interest in biopolymers as replacements for conventional durable fossil fuel-based materials reflects a global shift towards sustainable solutions^10^ driven by environmental concerns and regulatory pressures.^11^ Concerns about the accumulation of waste have heightened the focus on sustainable alternatives with more circular end-of-life options.

Starch, a natural polysaccharide derived from renewable agricultural resources such as corn, potato, and cassava, is widely regarded as a promising biopolymer for replacing fossil-fuel based materials, such as adhesives, films, and resins.^12–14^ Its inherent biodegradability, abundance, and low cost make it an attractive candidate for producing eco-friendly packaging, disposable items, and films.^13,15^ Moreover, the film-forming ability of starch, when processed through plasticization or blending, enables the creation of flexible, lightweight materials suitable for a range of applications. However, the thermoplastic character of starch presents significant challenges to replacing thermoset resins. Its hydrophilic nature leads to high moisture sensitivity, resulting in poor water resistance and limited durability under humid conditions.^16^ Starch-based plastics also exhibit brittleness and insufficient mechanical strength compared to conventional plastics,^17^ necessitating the use of plasticizers or blending with other polymers to improve flexibility and toughness. Additionally, processing starch requires specific conditions to avoid thermal degradation, and its compatibility with industrial-scale production processes remains an ongoing area of research. Despite these challenges, advancements in modification techniques and composite formulations continue to expand the potential of starch as a viable, sustainable alternative to traditional synthetic materials.^18^

On the other hand, lignin is a complex biopolymer found in plant cell walls, providing structural support and resistance to degradation. It is primarily composed of aromatic macromolecules derived from three monolignols: *p*-coumaryl alcohol, coniferyl alcohol, and sinapyl alcohol. These form hydroxyphenyl, guaiacyl, and syringyl units, which determine lignin's properties.^19^ Lignin, being one of the most abundant biopolymers on our planet, also exhibits potential to replace aromatic fossil fuel-based resources. Of the 100 million tons of lignin generated annually through biomass processing, only approximately 2% are commercially utilized^20^ for diverse applications such as binders, emulsifiers, and adhesives.^21^ Currently, the majority of lignin is still being burned in pulping plants for energy production. As lignin is a by-product of the pulping process and not the primary product, its quality and purity can be relatively low. The specific production method influences its composition, often resulting in a mixture of impurities, including carbohydrates, proteins, and inorganic salts.^22^ Moreover, the structural complexity and wide variability of lignin remain significant challenges for its direct use in application for high-value products. Although significant efforts have been made over the past few decades to characterize its structure, a clear understanding of its molecular composition has only recently emerged.^23^ Controlling lignin's multifunctional nature is a crucial step in its refinement and valorization. For this reason, chemical modification has been recognized as a key strategy to address these issues, primarily by enhancing chemical reactivity, reducing polymer brittleness, improving solubility in organic solvents, and facilitating the processing of lignin.^24–26^ Like polysaccharides, the modification of lignin relies on the presence of multiple hydroxyl groups, whether aliphatic or phenolic. Attempts to crosslink lignin to produce materials with higher bio-based content have been reported through esterification with diacids,^27^ acetal formation with aldehyde,^28^ and urethane formation with diisocyanate.^29^

Combining starch with lignin has been investigated as an effective strategy to address starch's deficient properties compared to fossil fuel-based materials, such as moisture sensitivity, thermal stability, and mechanical properties. Research has primarily focused on enhancing water resistance and decreasing the hydrophilicity of starch-based materials by integrating lignin into the starch matrix.^30^ Several attempts have been reported to combine these two polymers. The focus has been towards the development of starch blends, more specifically thermoplastic starch, in which lignin is incorporated to improve both the film properties and the resistance to degradation of the film.^30^ Indeed, lignin is a naturally occurring material with exceptional UV absorption capabilities, attributed to its aromatic structure and the presence of phenolic groups, ketones, and intramolecular hydrogen bonds.^31^ For example Majeed *et al.*^32^ tried to introduce lignin in urea cross-linked starch to improve coating properties. However, lignin's compatibility with other biopolymers and polymers, including starch, is limited due to its weak interfacial bonding, which often results in particle aggregation and phase separation. To overcome this issue, attempts to chemically bond starch and lignin have been made. Indeed, crosslinking of starch with lignin has been reported by using ammonium zirconium carbonate,^33^ glutaraldehyde,^34^ and urea after modifying both materials with aldehyde.^35^ More recently, Rashedi *et al.*^36^ cross-linked starch and lignin using a diepoxy crosslinker to improve the rheological and thermal properties of starch. The resin was produced through the formation of stable and resistant ether bonds, which prevent material degradation and enhance the recyclability of the bonded material.

Over the past years, research has increasingly been focused on integrating dynamic bonds into polymer structures, utilizing a click chemistry approach.^37,38^ These bonds can form and break in response to external stimuli, such as heat, pH changes, or light irradiation, enabling reversible cross-linking in polymers.^39^ This property leads to the creation of self-healing, debondable, and reprocessable materials. These architectures, known as covalent adaptable networks (CANs), can be either associative or dissociative, depending on the nature of bond formation.^40^ One of the most widely used reactions in CAN formation is the Diels–Alder (DA), a [4 + 2] cycloaddition between a diene and a dienophile, offering 100% atom efficiency. For bio-based applications, the DA cycloaddition between furan and maleimide groups, acting as diene and dienophile respectively, provides a versatile approach to form reversible bonds.^41^ This reaction offers several benefits, including its relatively low reaction temperatures around 60 to 70 °C for bond formation and 110 to 120 °C for the reverse reaction, which is well-suited to the thermal stability of starch and lignin.^42^ In response to this, numerous such as lignin, tannins, alginate, and various other polysaccharides.^43–45^ Indeed, several studies have employed the DA reversible reaction to create starch hydrogels, either by using a bismaleimide molecule to cross-link furan-modified starch or by reacting furan-modified starch with maleimide-modified starch.^46,47^ The focus of the past years has been on introducing DA in thermoset resins, such as polyurethanes and epoxies, to maintain good mechanical properties and enable recyclability.^48^ Nevertheless, little work has been done on developing fully bio-based reversible systems. The main interest in using DA reaction lies in the possibility of designing smart materials able to debond from each other or a given substrate, to debond back to initial components, to be reprocessed, and even self-healed.^49^ This enables an easier recycling of composite materials if used as an adhesive, and offering composites a longer service-life if used as a coating. This approach enhances the structural integrity and functional properties of starch and lignin, rendering them more suitable for advanced applications. The DA reaction, a type of click chemistry,^41,50^ is particularly advantageous due to its efficiency, mild reaction conditions, and eco-compatibility.^51^ We believe that by incorporating click chemistry, these biopolymers can be tailored to exhibit improved compatibility and advanced properties, paving the way for their use in innovative and sustainable materials. Indeed, by crosslinking a polysaccharides with a phenolic compound it is possible to create a material that benefits from both advantages of starch and lignin. To the authors knowledge such investigation has not been made previously.

In this study, we report the development of a novel temperature-responsive material by combining the properties of starch and lignin with the advantages of the furan–maleimide DA reaction. The present work reports a new one-pot esterification at room temperature of starch using a solvent as a catalyst. Potato starch (St) was modified using 6-maleimidohexanoic acid (6-MHA), and kraft lignin (KL) was selectively modified at its phenolic chain ends using furfuryl glycidyl ether, introducing pendant maleimide and furan groups, respectively. These modifications were confirmed using spectroscopic methods (^31^P and ^1^H NMR, UV-Vis Absorption, and FTIR). Finally, furan and maleimide-grafted starch (St 6-MHA) and lignin (KL-Fu) were combined to perform the DA reaction, leading to formations of gels that returned to a liquid state upon triggering the reverse (retro) DA reaction. Our green method combines high carbon efficiency and one-pot/*in situ* synthetic methodologies, which significantly reduce the need to clean intermediate products. This, in turn, lowers energy demands and minimizes the extensive use of organic solvents.

## 2 Materials

Potato starch (St), kraft lignin (KL), oxalyl chloride, 6-maleimidohexanoic acid (6-MHA), dimethylacetamide (DMAc), dimethylsulfoxide (DMSO), furfuryl glycidyl ether (FGE), sodium hydroxide (NaOH), hydrochloric acid (HCl), lithium chloride, 2-chloro-4,4,5,5-tetramethyl-1,3,2-dioxaphospholane (TMDP, 95%), chromium(iii) acetyl acetonate (Cr(acac)_3_, 97%), cholesterol (greater than 99%), deuterated DMSO (DMSO-d_6_), deuterated trifluoroacetic acid (TFA-d_1_, 99.5% atom D),were purchased from Sigma-Aldrich (Stockholm, Sweden). All other solvents were American Chemical Society (ACS) grade and used as received.

## 3 Synthetic strategies

### 3.1 One-pot modification of starch

#### 3.1.1 Preparation of acyl chloride

6-MHA was dissolved in DMAc. Then, 1 equivalent of oxalyl chloride was added dropwise under ice bath. After adding, the reaction was conducted at room temperature under an inert atmosphere of argon for 3 h. Gaseous by-products (HCl, CO and CO_2_) were allowed to escape the reaction mixture. At the end of reaction, the resulting acyl chloride was used without further purification.

#### 3.1.2 Modification of starch with acyl chloride in solution

3.2 g of oven dried St were placed in a round flask bottom containing 50 mL dry DMAc, the mixture was stirred at 140 °C for 1 h, then the temperature was lowered to 100 °C and 1 g of lithium chloride was added, the suspension instantly become transparent and the viscosity obviously rise to obtain a slurry. The solution was heated overnight to assess complete dissolution of St. The solution of St was added into the solution of acyl chloride in DMAc corresponding to 2 equivalent of hydroxyl group per anhydroglucose unit, and the reaction mixture was stirred at room temperature for 24 h under continuous flow of Argon. After reaction, the St was precipitated using methanol.

Then, the modified St was washed/centrifuged with methanol 3–5 times, and then freeze-dried.

#### 3.1.3 Functionalization of KL with furfuryl glycidyl ether

KL-Fu was synthetized based on previously reported method^23^ without particularly considering a green approach. (1 g) was dissolved in water containing NaOH (200 mg, 5 mmol, corresponding to 1 eq. of total acidic groups in KL, *i.e.* phenolic OH and COOH). After 1 h of stirring, FGE was added (1.5 eq. of KL phenolic OH) and the reaction mixture was stirred at 50 °C for 24 h. After cooling to room temperature and acidifying to pH 2 using 10% (v/v) aqueous HCl, the suspension was centrifuged to recover the precipitated KL. The functionalized KL was then washed 3 times with 50 mL acidified water (pH 2) and subsequently freeze-dried.

## 4 Characterization

### 4.1 Amylose content of St

The amylose/amylopectin content of the St samples was determined by a colorimetric method previously described.^52^ It consists of measuring the absorbance at 620 nm using ultraviolet-visible spectrophotometer (Lambda 35 UV/vis spectrometer, PerkinElmer, Waltham, USA) of a standard solution of pure amylopectin/amylose bound with iodine salt at different ratio to build a standard curve on the evolution of absorbance for the different amylose composition. The St amylose content was evaluated by the absorbance peak at 620 nm and compared with the standard curve. An amylose content of 17.1% was measured for St.

### 4.2 Spectroscopy

Spectrum Two FTIR (PerkinElmer, Waltham, USA) was equipped with an UATR Diamond accessory, which allows collection of FTIR spectra directly on a sample without any special preparation. The “pressure arm” of the instrument was used to apply a constant pressure (monitored by software) to the sample positioned on top of the diamond crystal to ensure a good contact between the sample and the incident IR beam. All FTIR spectra were collected at a spectrum resolution of 4 cm^−1^, with 32 scans over the range from 4000 to 450 cm^−1^.

All NMR spectra (^1^H, ^13^C, ^13^C DEPT, ^31^P, and 2D NMR spectroscopies) were recorded on a Bruker Avance III 600 MHz spectrometer (Billerica, USA). Samples were dissolved in DMSO-*d*_6_.

For ^31^P NMR spectroscopy, a 0.1 M solution of cholesterol in a pyridine/CDCl_3_ anhydrous mixture (1.6 : 1) (v/v) was used as internal standard, Chromium(iii) acetylacetonate was added into the internal solution as a relaxation agent. TMDP was used as phosphorylating agent, as described in standard protocols^53^ 128 scans were recorded with 10 s delay and a spectral width of 100 ppm (180–100 ppm).

For ^1^H NMR spectroscopic analysis, TMS was used as a reference. In this case, 16 scans were collected with 30 s delay. In a typical procedure for proper dissolution of St for ^1^H NMR analyses, 100 to 150 mg of St were suspended in 800 μL of dry DMSO-*d*_6_. The suspension was then shaken overnight on a bidirectional mixer at 80 °C and finally transferred to a 5 mm NMR tube for analysis at room temperature. When TFA-d_1_ was used, one to two drops were added to the solution just prior to measurement.

The degree of substitution (DS) of St-6-MHA was calculated by ^1^H NMR spectroscopy, similarly to a commonly reported procedure.^54^ It is based on the determination of the areas of the peaks assigned to grafted 6-MHA and those assigned to St backbone. After the addition of d_1_-TFA, each α-d-glucopyranose unit contains seven protons. However, since St always contains traces of water that d-TFA wasn't able to deuterated, only the peak corresponding to the proton of the St at 5.19 ppm was usable. The DS was thus calculated using the following equation:1
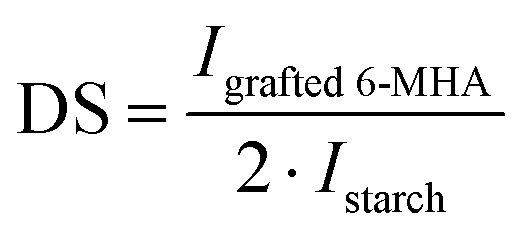
where *I*_grafted-6MHA_ and *I*_starch_ corresponds to the intensity of the protons from 6-MHA and to the intensity of the starch proton at 5.19 ppm, respectively.

### 4.3 Thermal stability

Thermogravimetric analysis (TGA) plots were carried out using a Mettler-Toledo TGA2 (Greifensee, Switzerland), under nitrogen with a flow rate of 40 mL min^−1^. Around 5 to 10 mg of samples were put in alumina pans. The samples were heated from 30 to 650 °C at a heating rate of 10 °C min^−1^.

### 4.4 Analysis of the DA and retro rDA reactions between St-maleimide and KL-furan

St-6-MHA and KL-Fu were dissolved in DMSO-*d*_6_ with an equimolar ratio of maleimide and furan functions. The reaction was carried out at 65 °C for 4 days to ensure the total formation of the DA adduct. The intensity of the peak at 7.62 ppm assigned to furan double bonds protons was integrated to compare the area of the peak before and after reaction.

The retro DA of St–KL sample was analyzed using a DSC3+ differential scanning calorimeter (Mettler Toledo, city, Switzerland). A mixture containing St-6-MHA and KL-furan with an equimolarity of functionalizing group in DMSO were heated at 65 °C for 3 days to ensure that the DA reaction occurred. Approximately 5 mg of this mixture were accurately weighed by a balance with a resolution of 0.00001 g and put into an aluminum crucible of 100 μL with the empty aluminum crucible (with lead pierced) as reference. The samples were heated from −30 to 220 °C and subsequently cooled down to −50 °C for 5 min at a rate of 30 °C min^−1^. Then, it was immediately heated from −50 to 200 °C at a rate of 30 °C min^−1^. The first heating curve was use for functionalized materials when only the second heating curve was analyzed for raw materials. Dry nitrogen gas flow of 10 mL min^−1^ was used to purge the furnace chamber of the DSC instrument.

Modified St and KL were dissolved in DMSO with an equimolar ratio of maleimide and furan functions. DMSO was chosen as a solvent to minimize solvent evaporation during the measurement. The storage dynamic modulus (*G*′) the loss modulus (*G*′′), and viscosity were measured at 65 °C for 600 min using a rheometer (Haake Mars 60, Waltham, USA), equipped with a C35 1°/Ti measuring geometry at an oscillation frequency of 1 Hz. The sample was heated using a pelletier and temperature controller (MTMC Mars 60, Waltham, USA) and kept at a constant temperature using a water bath/circulator (Accel 500 LT, Waltham, USA).

### 4.5 E-factor

E-factor was calculated using the following equation:2
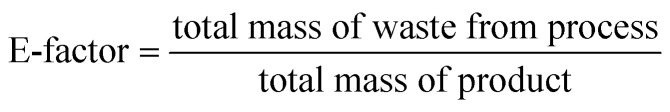


By definition it takes into account waste such as byproducts of the reaction, unreacted reagents and solvent losses (anything else than the product of interest that can't be recovered for reuse). In the starch esterification process, we considered that potential wastes are the following:

- Lithium chloride used in starch dissolution.

- CO, CO_2_ and HCl produced during acyl chloride formation.

- Excess of acyl chloride during starch esterification.

- DMAc used as solvent and methanol used to washed esterified starch.

Considering that the only wastes remaining in the solvent after starch modification is unreacted acyl chloride and starch residues, it is possible to recover DMAc and methanol by distillation. Thus DMAc and methanol were not included in E-factor.

## 5 Results and discussion

### 5.1 Analysis of the synthesized St-maleimide

**Scheme 1:**
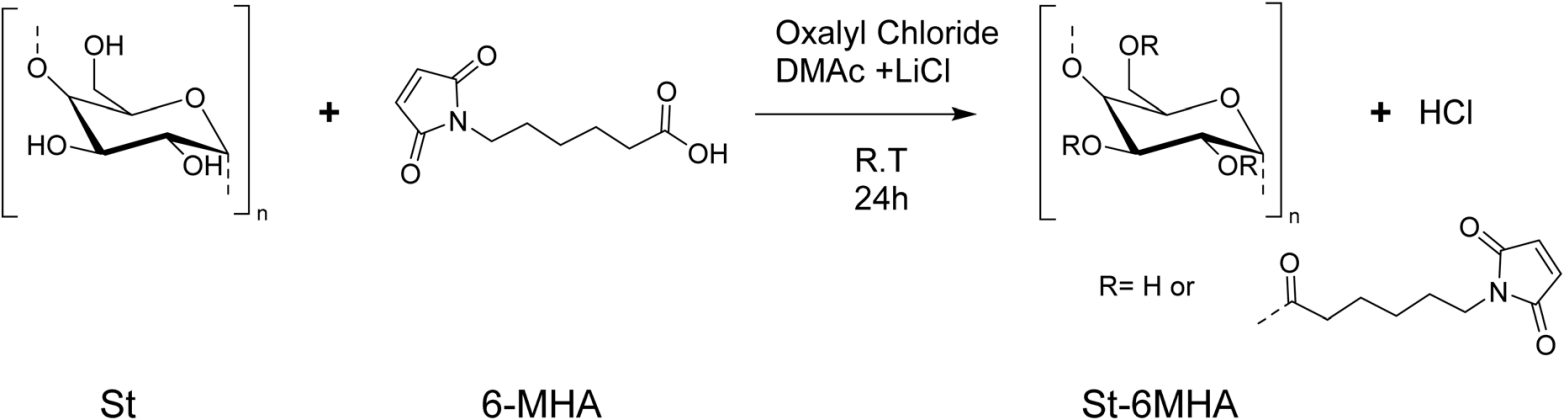
Synthesis of St 6-MHA.

The novelty of this esterification reaction (Scheme 1) is that it is performed in one pot at room temperature without the use of a harmful catalyst. We believe that the solvent also acts as a catalyst, similar to dimethyl formamide^55^ and can be recovered at the end of the reaction. An E-factor of 1.2 was calculated under the assumption that both DMAc (b.p. 165 °C), used as the reaction solvent, and methanol (b.p. 64.7 °C), used for washing the final product, can be readily recovered through distillation. Consequently, the only likely residues in the process are LiCl, unreacted 6-MHA (b.p. 407 °C) and either native starch or St-6MHA. If we make the common assumption that 10%^56^ of the solvents (DMAc and methanol) is lost, the E-factor raised up to 2.4. This raise the necessity for this reaction to be considered green to effectively recycle solvent.

**Fig. 1:**
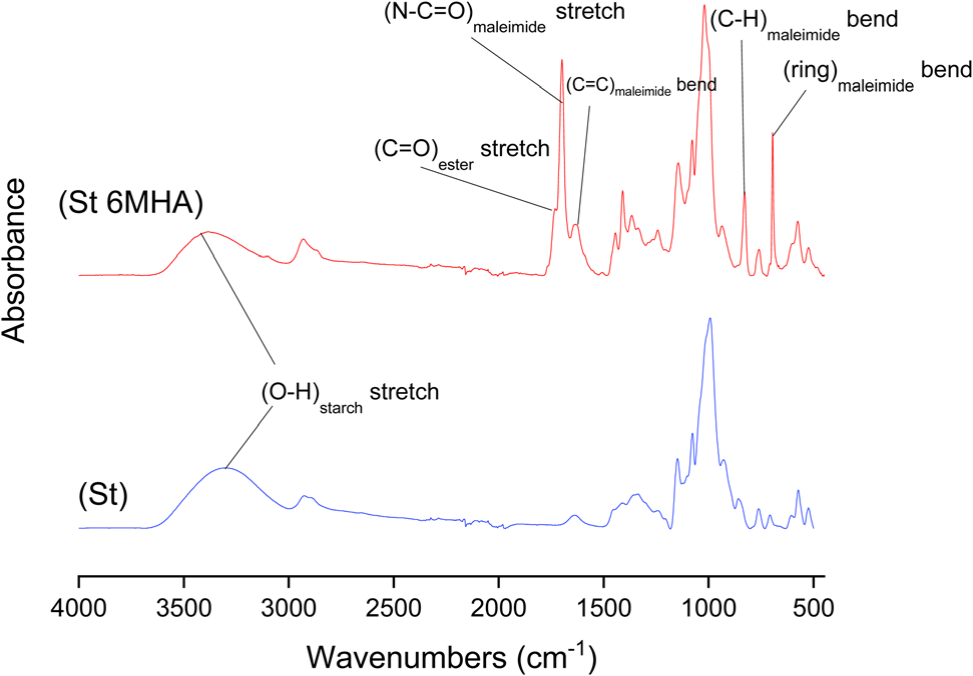
FTIR absorbance spectra of unmodified St and 6-MHA grafted St.

Prior to St modification, 6-MHA was turn into its corresponding acyl chloride to increase its reactivity towards St hydroxyl groups. The reaction was carried under inert atmosphere to avoid the reaction of water present in the air with the newly form acyl chloride. Former studies reported the direct use of the synthetized acyl chloride without further purification.^23,57^ We believe that it is hard to isolate the product and analyze it, as conventional FTIR is challenging to perform without hydrolyzing acyl chloride^23^ because of the moisture present in the air. NMR has been done previously by other researchers.^42^ Indeed, by evaporation of the solvent under vacuum followed by dissolution in deuterated solvent under inert atmosphere using septum, it is possible to transfer the product in NMR tube without any contact with the air. In order to minimize hydrolysis (potentially decreasing esterification yield), the acyl chloride was directly used without further purification, and the St solution in DMAc was added into it. The raw and modified St were analyzed by FTIR (Fig. 1).

Bands corresponding to the grafted 6-MHA St were observed at 1696, 1624 and 826 cm^−1^ respectively, attributed to maleimide N–C=O stretching, C=C bending and C–H ring bending. A band at 1740 cm^−1^ was attributed to ester C=O stretching confirming the esterification. Moreover, consumption of the hydroxyl after esterification was shown by the decrease of intensity of St hydroxyl groups stretching corresponding band around 3380 cm^−1^. These results are consistent with previous work found in the literature.^58,59^

^1^H and ^13^C NMR of native (unmodified) St and St 6-MHA were recorded and peaks were assigned in details to confirm the success of the modification before performing the DA reaction. In order to confirm ^1^H and ^13^C NMR's assignments, 2D NMR of native and modified St was, ^1^H–^13^C HSQC and HMBC spectra can be found in SI (Fig. S2–S5).

**Fig. 2:**
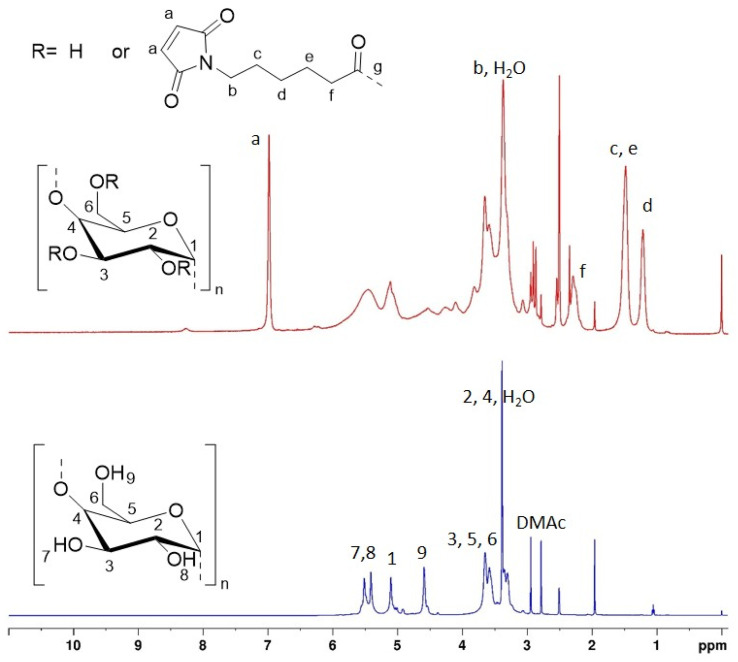
^1^H NMR spectra of unmodified St and 6-MHA grafted St.

**Fig. 3:**
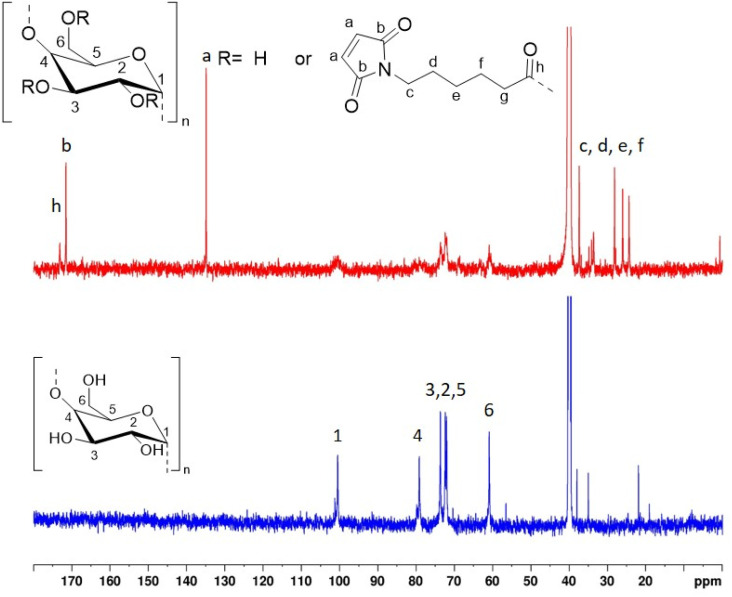
^13^C NMR spectra of unmodified St and 6-MHA grafted St.

The presence of maleimide groups grafted onto St was confirmed through ^1^H (Fig. 2) and ^13^C (Fig. 3) NMR. The ^1^H NMR spectrograms show peaks corresponding to the protons of grafted 6-MHA St peak at 6.90 ppm, attributed to the protons on the maleimide ring. This signal was used to determine the degree of substitution (DS) after d_1_-TFA addition, 0.97 ± 0.02. In theory, the addition of d_1_-TFA enables to exchange OH protons with TFA-d_1_ deuteron leading to shift all peaks corresponding to starch hydroxyl and water to a single peak around 8 ppm. Thus, allowing to simplify ^1^H NMR spectrum in order to use St backbone protons to determine DS. This method was described by Morinval *et al.*^42^ in order to calculate the degree of substitution of amylomaise St. Nonetheless, there is still traces of water trap into starch structure that doesn't exchange is proton with TFA-d_1_. Indeed, unlike potato St, amylomaise has a high amylose content, and thus less crystalline, and be easier to solubilize and get rid of water into the St core. For this reason, only the peak at 5.19 ppm corresponding to one of the St proton was clear enough to be use in this purpose. Spectra of St-6-MHA in DMSO-*d*_6_ are given in the SI.

On the ^13^C NMR spectrum (Fig. 3), peaks between 171.5 and 173.2 ppm respectively corresponded to (C=O) carbons of maleimide and ester, indicating successful esterification.

On both ^1^H and ^13^C NMR spectra of St 6-MHA the peaks corresponding to the St native structure appeared broader and flattened. This decrease in peaks intensity is due to partial esterification of St, increasing the number of different equivalent groups of protons, and thus broadening the peak range while lowering intensity.^60^ High scans number was required to make those peaks appeared on ^13^C NMR as ^13^C NMR is more sensitive to neighboring atoms than ^1^H NMR and has a lower natural abundance of ^13^C.

By integrating data from both 1D and 2D NMR spectroscopy, the observed peaks were successfully assigned to their respective hydrogen and carbon atoms. HMBC and HSQC of native St was performed (Fig. S2 and S3) to differentiate peaks from St and 6-MHA. The structure determination of St 6-MHA through HSQC (Fig. S5) and HMBC (Fig. S4) further confirmed the esterification. HSQC (Fig. S5) displayed peaks corresponding to the ring carbons and hydrogens of St when HMBC mostly shown the peaks corresponding to grafted FGE.

### 5.2 Analysis of the synthesized lignin-furan

**Scheme 2:**
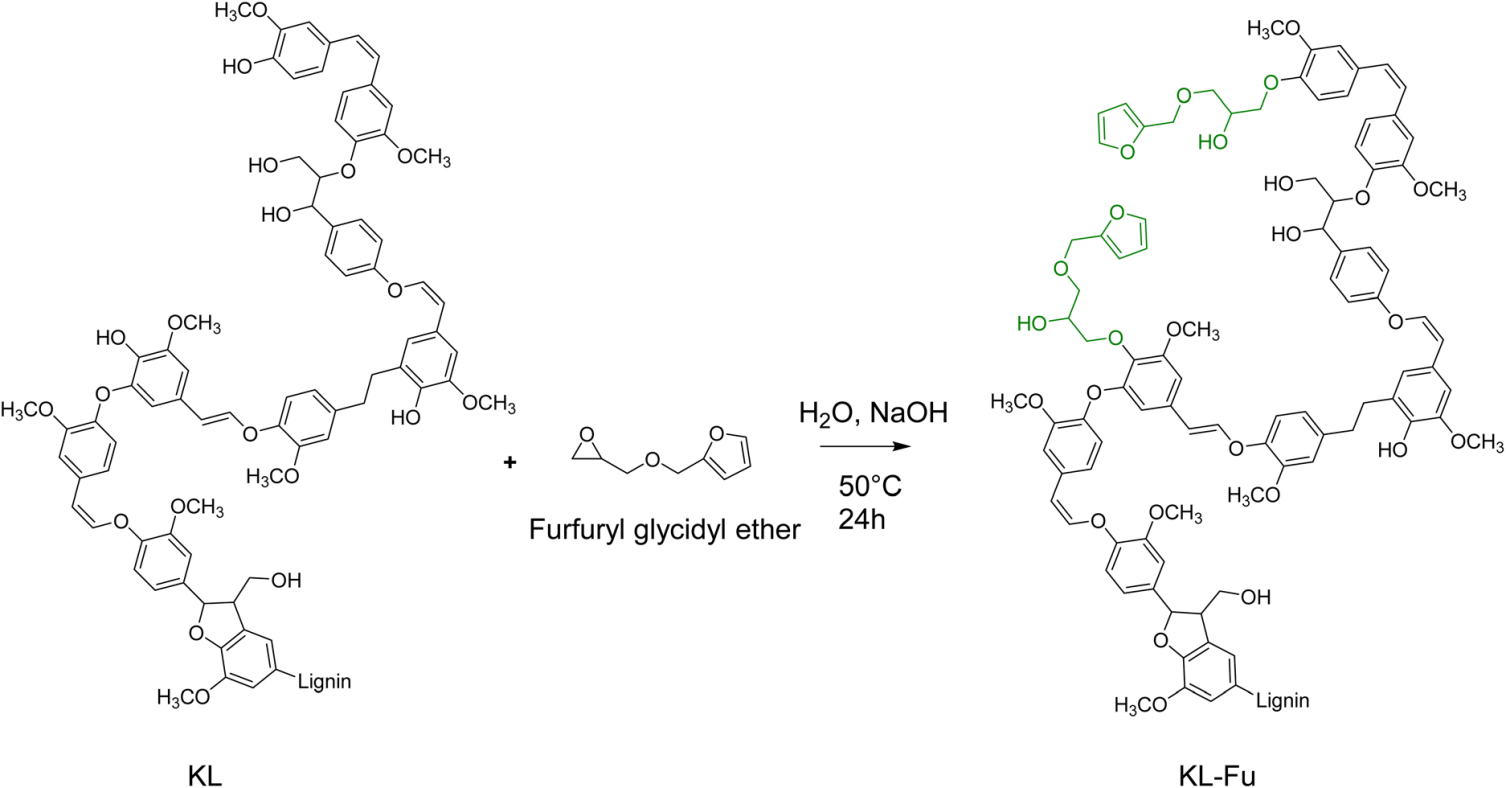
Synthesis of KL-Fu.

KL was functionalized with FGE to introduce furan rings mostly at the phenolic chain ends, as illustrated in Scheme 2. The reaction was conducted in water with a stoichiometric amount of NaOH, calculated based on the total acidic groups in KL (both carboxyl and phenolic hydroxyl groups). Under these conditions, only carboxyl and phenolic OH groups were deprotonated, while aliphatic OH groups remained unaffected due to their significantly higher p*K*_a_. As a result, phenolate ions acted as the reactive species, while carboxyl groups were inactive as carboxylates.

**Fig. 4:**
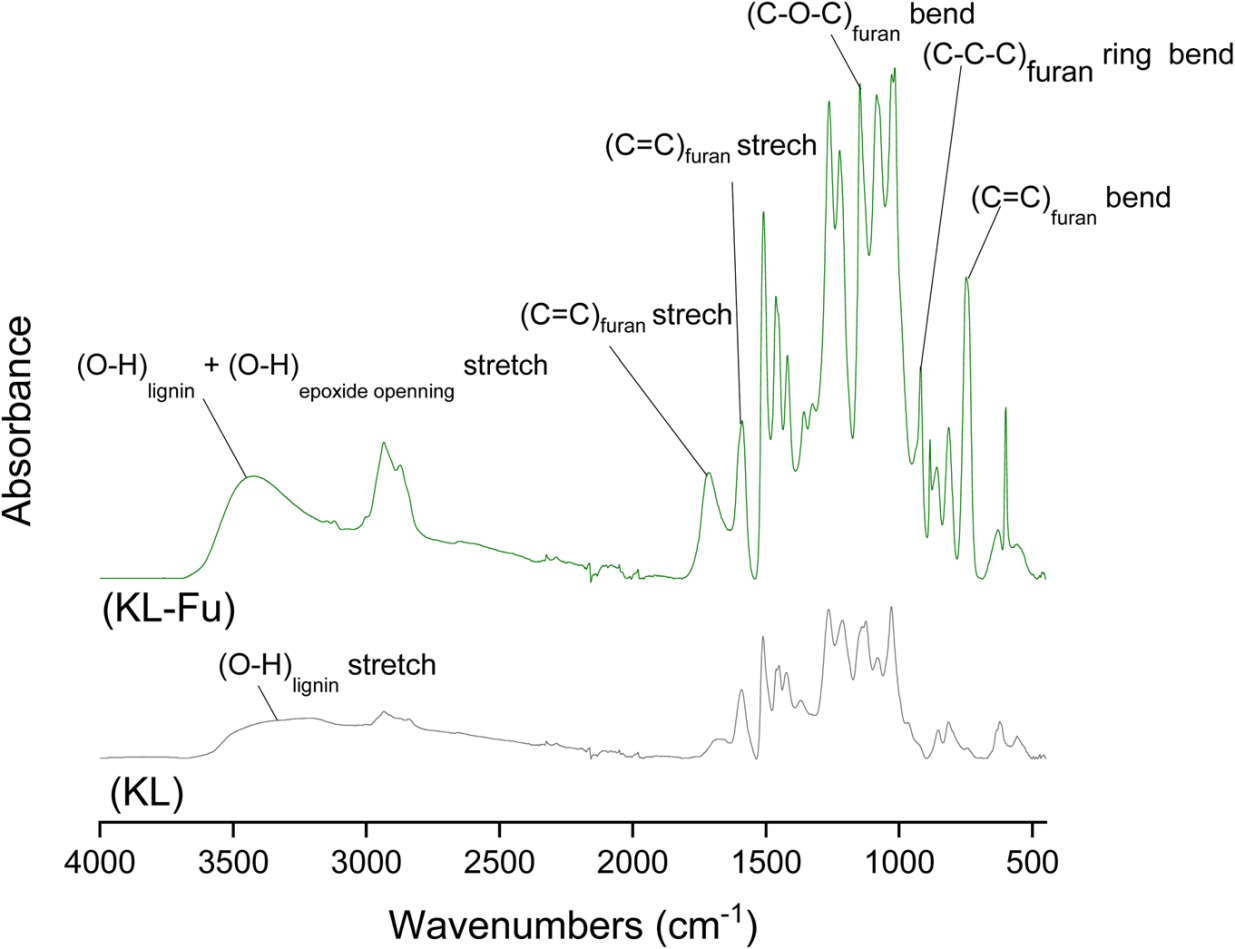
FTIR absorbance spectra of unmodified KL and furan grafted KL.

FTIR spectra of native KL and furan-grafted KL (KL-Fu) were recorded (see Fig. 4). Bands at *ν* = 1711, 1588, 918 and 750 cm^−1^ were attributed to furan C=C stretching, C=C stretching, furan ring bending and C=C bending, respectively.^23^ The furan grafting also cause an increase of intensity of the broad band between 3000 and 3600 cm^−1^, corresponding to O–H stretching due to the epoxide ring opening leading to the formation of aliphatic hydroxyl groups.

^1^H and ^13^C NMR KL and KL-Fu were recorded and peaks were assigned to confirm the reaction. ^1^H and ^13^C NMR spectra of furfuryl glycidyl ether were also recorded to help in assignment of new peaks (Fig. S10).

**Fig. 5:**
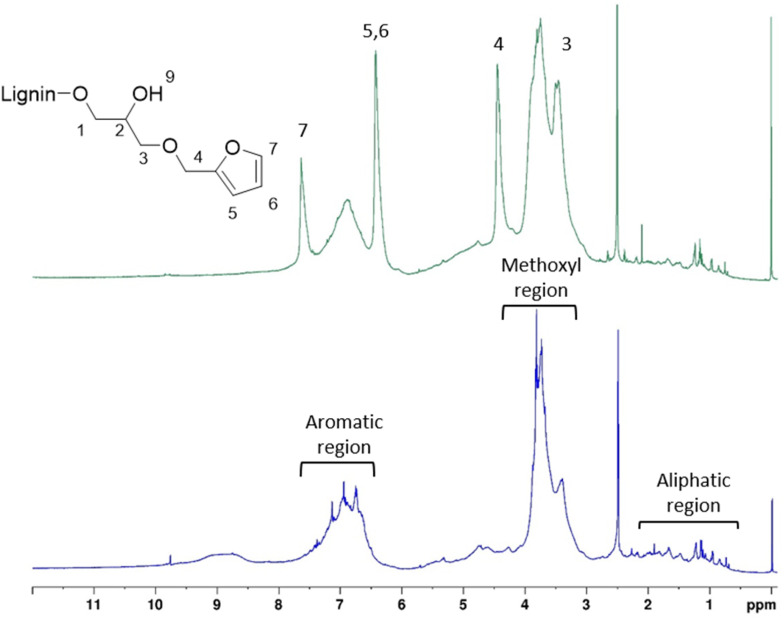
^1^H NMR spectra of KL and furan grafted KL.

**Fig. 6:**
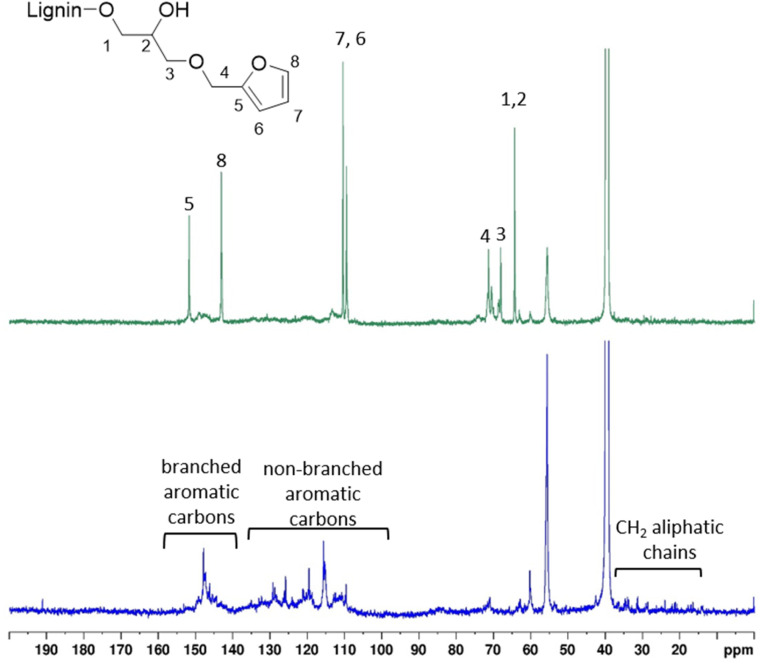
^13^C NMR spectra of unmodified KL and furan grafted KL.

The presence of maleimide groups grafted onto St was confirmed through ^1^H (Fig. 5) and ^13^C (Fig. 6) NMR. The ^1^H NMR spectrograms show peaks corresponding to the protons of grafted 6-MHA St peak at 6.90 ppm, attributed to the protons on the maleimide ring.

The ^1^H NMR spectra of the functionalized KL (Fig. 5), revealed two new peaks at 6.40 and 7.60 ppm, corresponding to the hydrogen atoms of the furan ring. Moreover, two peaks around 3.20 and 4.20 ppm corresponding to protons of the carbon chain of FGE could be observed.

The grafting of furan moieties onto KL was further confirmed through ^13^C NMR (Fig. 6), and clear peaks associated to FGE after ring opening of the epoxide could be distinguished from broad and less intense peak from KL. Indeed, peaks corresponding to carbons of the furan ring were identified at 110, and 142 ppm. The peaks of the remaining carbon of FGE were present between 60 and 70 ppm (Fig. 6).

**Fig. 7:**
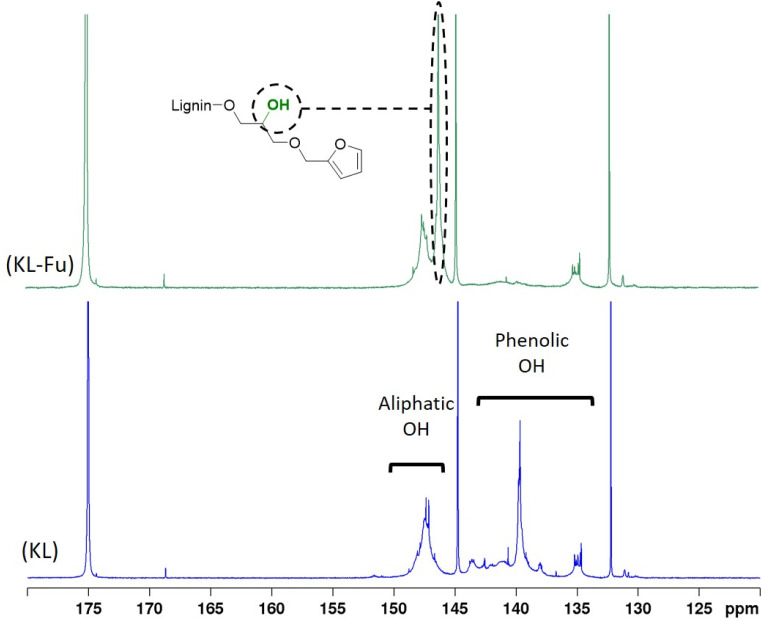
^31^P NMR spectra of unmodified lignin and furan grafted lignin.

As illustrated in Scheme 2, the epoxide ring opening generates a new aliphatic hydroxyl group, leading to an increase in aliphatic OH content. This reaction results in the appearance of a new peak with high intensity in the ^31^P NMR spectrum at 146 ppm, which is distinct from the original KL aliphatic OH signal, consistent with previous observations.^23^ Indeed, 3 distinct area characteristic to KL ^31^P NMR spectra: 134 to 136 ppm (carboxylic acid), 137 to 145 ppm (phenolic) and 145 to 150 ppm (aliphatic hydroxyl) are shown in Fig. 7.^53^ Moreover, 3 sharp peaks with high intensities are displayed in ^31^P NMR spectrum at 175, 144 and 132 ppm. They can be respectively attributed to the excess of TMDP, the cholesterol (internal standard) and the hydroxylated-TMDP. The presence of these peaks confirmed the sample complete derivatization.^53^

As it could be seen on NMR spectra of St-6MHA, the peaks corresponding to the KL native structure appeared broader and flattened. This decrease in peaks intensity is due to partial modification of KL, increasing the number of different equivalent group of protons and thus broadening the peak range while lowering intensity. High scans number was required to make those peaks appeared on ^13^C NMR as ^13^C NMR is more sensitive to neighboring atoms than ^1^H NMR and has a lower natural abundance of ^13^C.

The furan content of KL-Fu was measured through quantitative ^31^P NMR following the procedure reported by Argyropoulos *et al.*^53^ resulting as 2.30 mmol g^−1^. This result aligned with previous work reported by Duval *et al.*^23^ in which it was found that the total phenol content can react in the presence of a slight excess of FGE.

### 5.3 Analysis of the DA and rDA reactions between St-maleimide and KL-furan

**Scheme 3:**
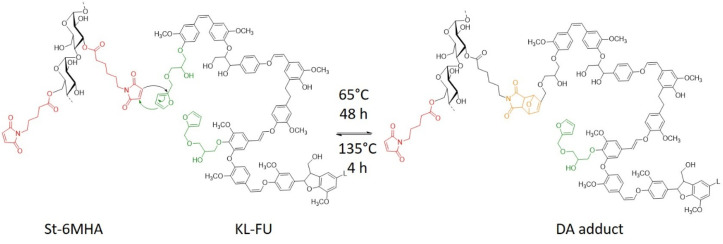
St-6-MHA/KL-Fu DA network.

The formation of DA network was investigated as we believe that such networks might show potential interest in the development of thermally responsive material such as on demand debonding adhesive. Moreover, this reversible network benefit from the green aspect of the DA reaction, which provides a 100% atom economy, through click-chemistry (Scheme 3).

In order to demonstrate the cross-linking between the modified materials *via* DA cycloaddition, St 6-MHA and KL-Fu were mixed in a 1 : 1 maleimide to furan ratio and dispersed in DMSO to create a liquid with very low viscosity, facilitating observation of gel formation as the modified starch form a gel by itself at high concentration. This mixture was heated to 65 °C and stirred overnight. The gel formation was observed, and later disassembled upon heating to 135 °C for 2 h. A darker solution was obtained. When the mixture was too dilute, a phase separation between the gel formed and the excess of solvent could be observed.

**Fig. 8:**
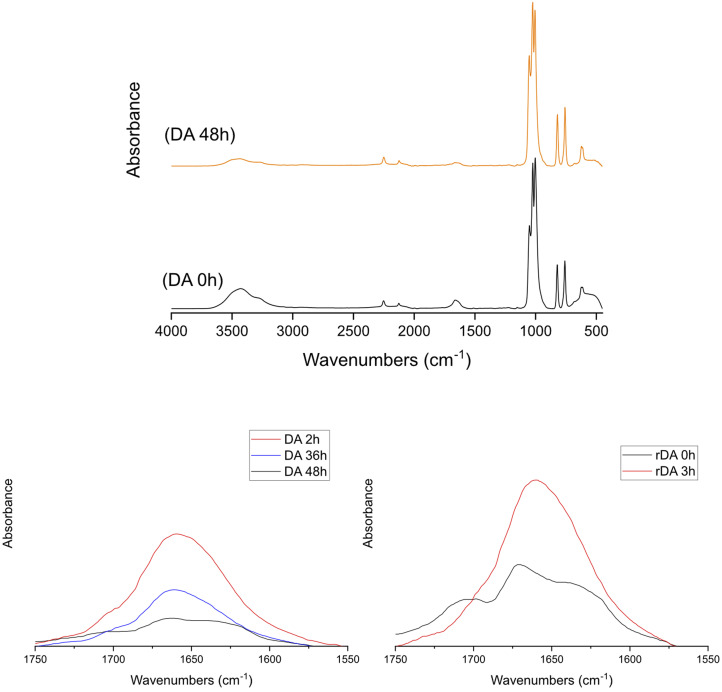
FTIR absorbance spectra of St-6-MHA/KL-Fu mixture before and after heating at 65 °C for 48 h.

To further confirm the forward and retro DA reactions, KL-Fu and St 6-MHA dispersed in DMSO and consecutively heated at 65 °C for 2 days and 135 °C for 3 hours, this sequence was repeated 2 times and FTIR spectra were recorded at regular time points. The decrease of intensity of the band around 1640–1680 cm^−1^ corresponding to the maleimide C=C bending, showed the formation of the DA adduct.^61^ As can be seen on Fig. 8, the ratio between the intensity of this band and the band corresponding to the DMSO clearly confirmed the consumption of maleimide in the forward reaction and regeneration in the retro reaction.

**Fig. 9:**
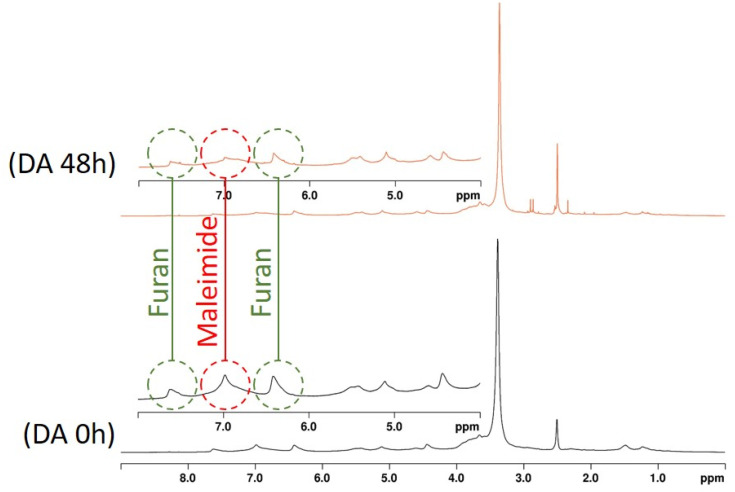
^1^H NMR spectra of St-6-MHA/KL-Fu mixture before and after heating at 65 °C for 48 h.

The analysis of DA reaction was also confirmed by ^1^H NMR (Fig. 9), and the modified St and KL were dissolved in DMSO-*d*_6_ at low concentration to avoid gel formation to obtain a clear signal. The spectrum revealed a decrease of the peak intensity at 7.62 ppm corresponding to the furan double bonds. This observation is consistent with the disappearance of the furan double bonds during the formation of DA adduct. A 70% conversion was estimated by using peaks at 7.00 related to maleimide consumption aligning with the literature.^42^

**Fig. 10:**
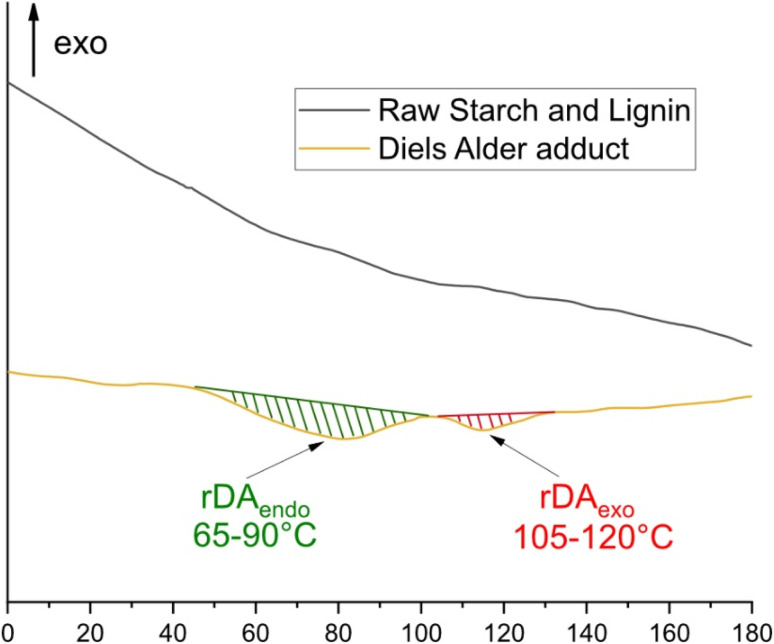
DSC thermogram of St-6-MHA/KL-Fu mixture after heating at 65 °C for 48 h.

The decomposition of DA adduct was investigated through Differential Scanning Calorimetry (DSC). A mixture of raw St and KL as well as the DA adduct were placed in 100 μL crucible and heated from 0 to 180 °C, and the obtained thermograms are presented in Fig. 10. In order to prove that the peaks observed in Fig. 10 do not correspond to individual behavior of St, St 6-MHA, KL and KL-Fu, their DSC thermograms were recorded and can be found in supported information (Fig. S6 and S7). Moreover, to show the stability of all materials under this range of temperature, TGA and DTG thermograms of St, St 6-MHA, KL and KL-Fu were recorded between 30 and 630 °C (Fig. S8 and S9). As reported in previous work,^59^ the thermal stability of St (Fig. S8) seems to be slightly better after modification. Moreover, the TGA thermograms of St showed a two-step degradation corresponding to the degradation of St backbone followed by the degradation of 6-MHA further confirming the success of esterification. Same observations were made for KL (Fig. S9). It was concluded that St 6-MHA and KL-Fu could both support the thermal condition of forward and retro-Diels–Alder reaction. This enabled to ensure preventing the degradation of renewable feedstocks and maintaining the functional performance of biobased components.

While the DSC thermograms of initial materials (St mixed with KL) do not show the presence of any peaks (aligning with TGA's observations), the thermograms of DA adduct show the presence of two broad endothermic peaks around 80 and 110 °C. These two peaks correspond to the retro DA of the two DA adducts diastereoisomer. Indeed, in the DA reaction, two possible stereoisomeric products can be formed: the *endo* and *exo* products. The *endo* product arises when the electron-withdrawing group of the dienophile is oriented towards the diene, benefiting from secondary orbital interactions. This makes the *endo* pathway kinetically favored, meaning it forms more quickly due to a lower activation energy. In contrast, the *exo* product is often the thermodynamically more stable form but forms more slowly. As a result, the *endo* product is expected to dominate, especially at lower temperatures with bulky substrates.^62^ It is well known that the *exo* diastereoisomer cleaved after the *endo* hence the higher temperature peak can be assigned to the *exo* diastereoisomer.^42^

The enthalpy energy of the contribution from *endo* and *exo* peaks was measured using star-e software (Metler Toledo, Greifensee, Switzerland), and it was calculated that the *endo* product has a significantly higher contribution with a factor 2.2. In the end, the DSC analysis enabled to further confirm the reversibility of the DA adduct.

The rheological behavior of a mixture of raw St and KL as well as St 6-MHA and KL-Fu was studied as a trial to confirm the forward DA reaction. It has to be noticed that, after 10 h at 65 °C, both mixtures had a completely different aspect. While the mixture of raw materials appeared as a slurry viscous solution, the mixture of modified materials appeared instead as a thin film coating the surface of the metal base of the rheometer apparatus.

**Fig. 11:**
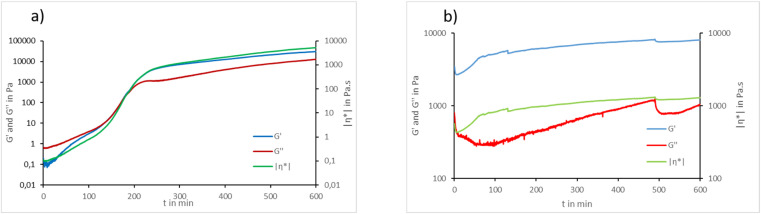
Rheological analysis of a mixture of raw St and raw KL (a) and a mixture of St 6-MHA and KL-Fu (b) at 65 °C for 600 min.

The graphs in Fig. 11 show the evolution of storage modulus (*G*′), loss modulus (*G*′′) and the modulus of complex viscosity |η*| of a mixture containing St and KL (11a) and a mixture containing St 6-MHA and KL-Fu (11b). When the DA material is heated to 65 °C, which corresponds to the forward DA reaction and possibly the gel-point temperature its loss modulus does not surpasses the elastic modulus, indicating a sol–gel behavior.^63^ On the other hand, for the native materials mix, when heated to 65 °C in the same conditions, its viscous modulus surpasses the elastic modulus, indicating a liquid-like sol behavior.^63^ The mixture of St and KL displayed an increase in both moduli and viscosity along time. Starch is known to exhibit shear-thickening behavior, wherein viscosity increases with shear frequency. Although the experimental frequency was held constant throughout our measurements. It was reported that shear-induced physical crosslinking^64^ can happen in St dissolved in 90 : 10 DMSO : water solution. This can be seen as the formation of a highly entangled network of St itself as well as between St and KL. We also believe that a prolonged heating can induce solvent evaporation, leading to an increased concentration of starch and, consequently, a rise in viscosity. Moreover the fact that *G*′ is higher than *G*′′ indicated that the mixture tends to behave like a solid (elastic) rather than a liquid (viscous). We believe that upon heating, the starch and lignin components have the opportunity to interact *via* intermolecular hydrogen bonding. The hydroxyl groups of starch may form hydrogen bonds with both aliphatic and phenolic OH groups present in KL. As heating might cause a partial loosening of starch's granular structure (increase of chains distance with temperature),^65^ improving its accessibility and increasing the likelihood of interfacial contact with lignin. This enhanced intermolecular bonding contributes to a denser network structure, which is expected to result in *G*′ being higher than *G*′′. Moreover, the starch gelatinization has been found to be dependent on hydrogen bond density in the solvent.^66^ Adding KL to DMSO could decrease the volumetric density of intermolecular hydrogen bonds leading to an increase in St gelatinization temperature.

On the graph of DA (Fig. 11b) we can see twice the breakage of the materials resulting in two consecutive drastic drops after 120 and 480 min. This shows the different state of both materials. On one side, we have a viscous solution slowly turning into entangled network. On the other side we have a fast curing cross-linked gel. We believe that the curing is more efficient in the rheometer set up. The small sample thickness,^67^ which is less than 1 mm, resulting in a higher surface-to-volume ratio in contact with the plates. This geometry allows heat to be transferred more rapidly and uniformly throughout the material, potentially resulting in faster curing.

## 6 Conclusions

In conclusion, a new thermally responsive and sustainable macromolecular system was successfully developed. Efficient synthetic strategies for the functionalization of potato St and KL were established. St was first esterified through a one-pot synthesis, and the grafting of maleimide groups onto the starch backbone was clearly confirmed by FTIR and NMR spectroscopies. On the other hand, KL was modified with furfuryl glycidyl ether, as demonstrated by FTIR, ^1^H, ^13^C, and ^31^P NMR spectroscopies. The two counterparts were conjugated through the DA reaction between the furan and maleimide functionalities of the two modified biopolymers. This reaction was confirmed through various spectroscopic and thermal analysis methods. Subsequently, the reversibility of the DA-conjugated network was examined thermally, and it was found that the retro DA reaction is possible through heating at 135 °C. Indeed, the adduct decomposition was shown by DSC and the reversibility was followed with the evolution of maleimide characteristic band by FTIR through successive heating cycles at 65 (DA) and 135 °C (rDA). By harnessing the advantages of the furan–maleimide DA reaction, this study highlights a sustainable approach on developing advanced biopolymer systems. The efficient synthetic methodologies reduce the need for intermediate cleaning steps, thus lowering energy demands and minimizing the use of organic solvents. This thermally responsive macromolecular system not only addresses environmental challenges posed by fossil fuel-derived materials but also contributes to the advancement of eco-friendly materials with enhanced functional properties.

## Author contributions

Conceptualization R. P., S. A. and V. S.; methodology and experiments design, R. P., V. S.; validation, R. P. and S. A.; analysis, V. S.; investigation, V. S.; resources, S. A.; writing—original draft preparation, V. S.; writing—review and editing, R. P., V. S. and S. A.; visualization, V. S. All authors have read and agreed to the published version of the manuscript.

## Conflicts of interest

The authors declare no conflict of interest.

## Supplementary Material

RA-015-D5RA02344K-s001

## Data Availability

The data presented in this study are available from the corresponding author upon request. Supplementary information includes: S1. ^1^H NMR spectra of raw potato starch (blue) and St 6MHA (red) in DMSO-d_6_ after adding TFA-d_1_, S2. HMBC 2D NMR spectrum of raw potato starch in DMSO-d_6_, S3. HSQC 2D NMR spectrum of raw potato starch in DMSO-d_6_, S4. HMBC 2D NMR spectrum of St-6MHA in DMSO-d_6_, S5. HSQC 2D NMR spectrum of St-6MHA in DMSO-d_6_, S6. DSC thermogram of unmodified and modified Starch, S7. DSC thermogram of unmodified and modified Lignin, S8. TGA and DTG thermograms of unmodified and modified Starch, S9. TGA and DTG thermograms of unmodified and modified Lignin, S10. ^1^H and ^13^C NMR spectra of Furfuryl glycidyl ether in DMSO-*d*_6_. See DOI: https://doi.org/10.1039/d5ra02344k.
