# Supplementary material for: One-pot synthesis of thermally reversible materials using maleimide-polysaccharide and furan-lignin derivatives
Source: RSC Adv. 2025 Aug 8;15(34):28255–68. doi: 10.1039/d5ra02344k (PMC12376768; doi:10.1039/d5ra02344k)
Supplement: RA-015-D5RA02344K-s001 [file RA-015-D5RA02344K-s001.pdf]

Supplementary Information (SI) for RSC Advances.
This journal is © The Royal Society of Chemistry 2025

One-Pot Synthesis of Thermally reversible Materials Using Maleimide-Polysaccharide and Furan-Lignin Derivatives

Valentin Silveira[a], Raffaello Papadakis, and Stergios Adamopoulos[a,✉]

[a]*Department of Forest Biomaterials and Technology, Swedish University of Agricultural Sciences, Vallvägen 9C, 756 51 Uppsala, Sweden*

✉Stergios Adamopoulos, stergios.adamopoulos@slu.se, +46 73-572 27 86

Supporting Information

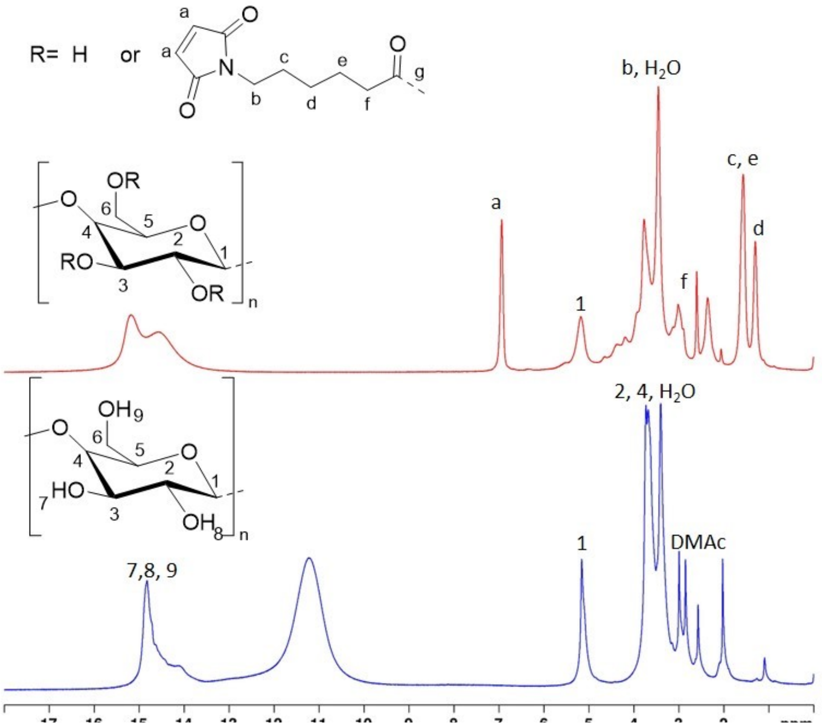

Figure S1. ^{1}H NMR spectra of raw potato starch (blue) and St 6MHA (red) in DMSO-d_6 after adding TFA-d_1

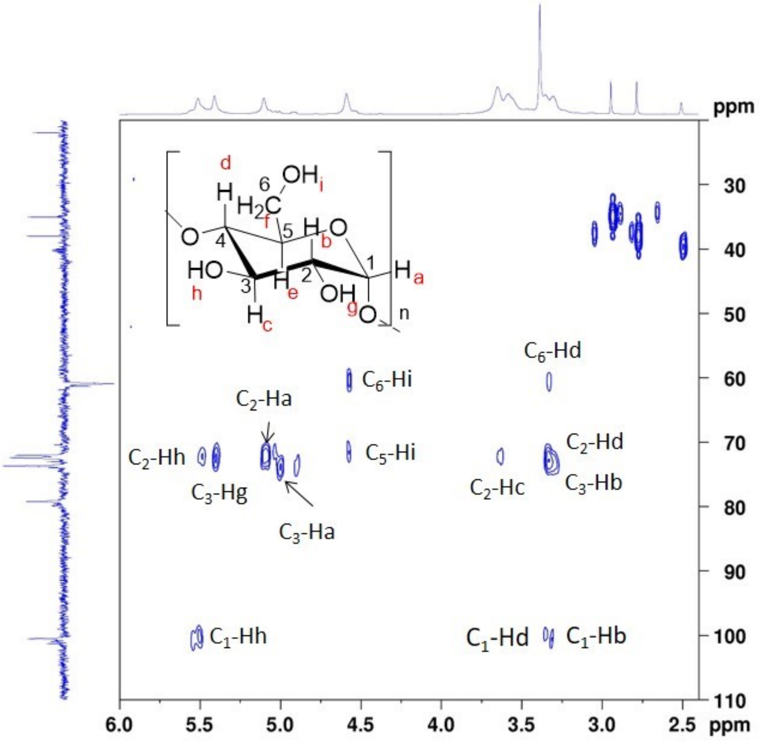

Figure S2. HMBC 2D NMR spectrum of raw potato starch in DMSO-d_6

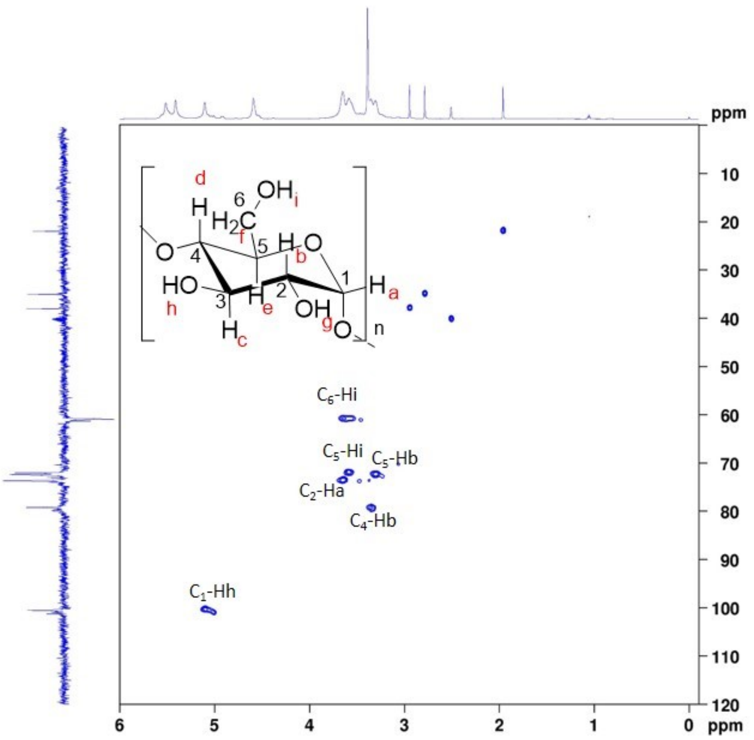

Figure S3. HSQC 2D NMR spectrum of raw potato starch in DMSO-d_6

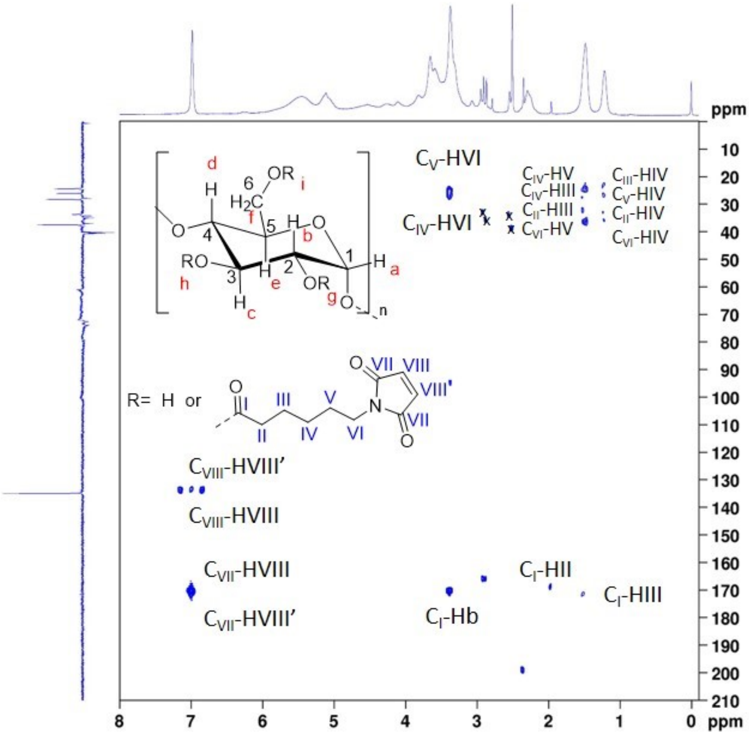

Figure S4. HMBC 2D NMR spectrum of St-6MHA in DMSO-d$_6$

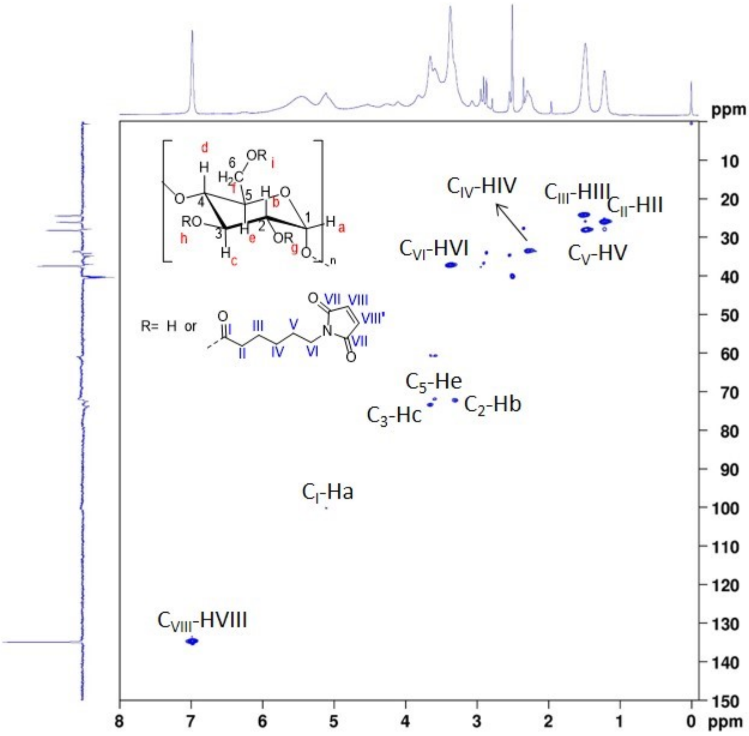

Figure S5. HSQC 2D NMR spectrum of St-6MHA in DMSO-d$_6$

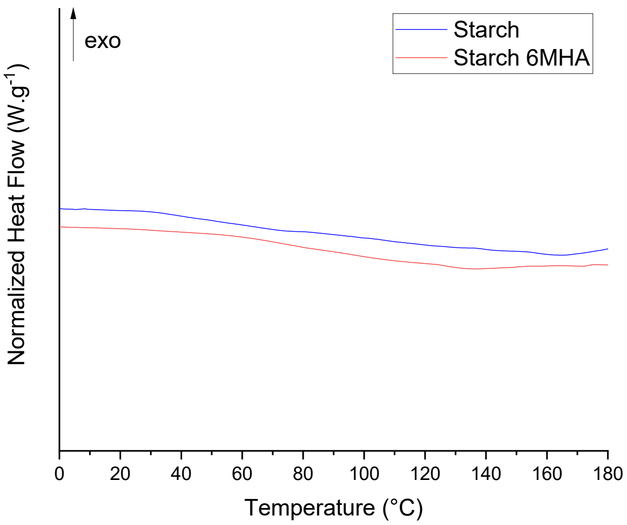

Figure S6. DSC thermogram of unmodified and modified Starch

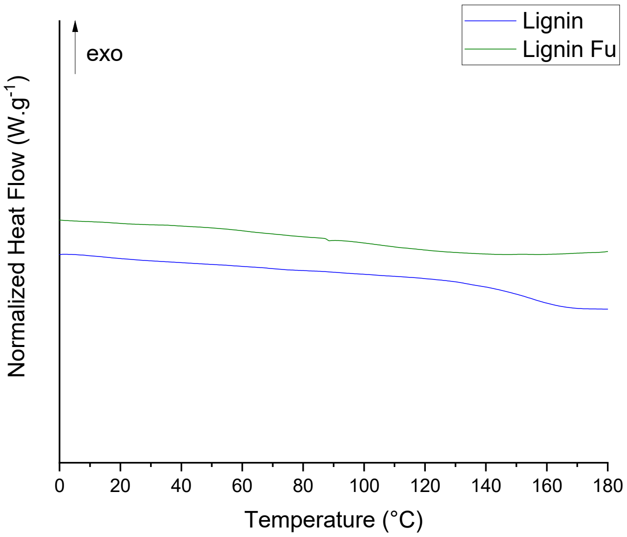

Figure S7. DSC thermogram of unmodified and modified Lignin

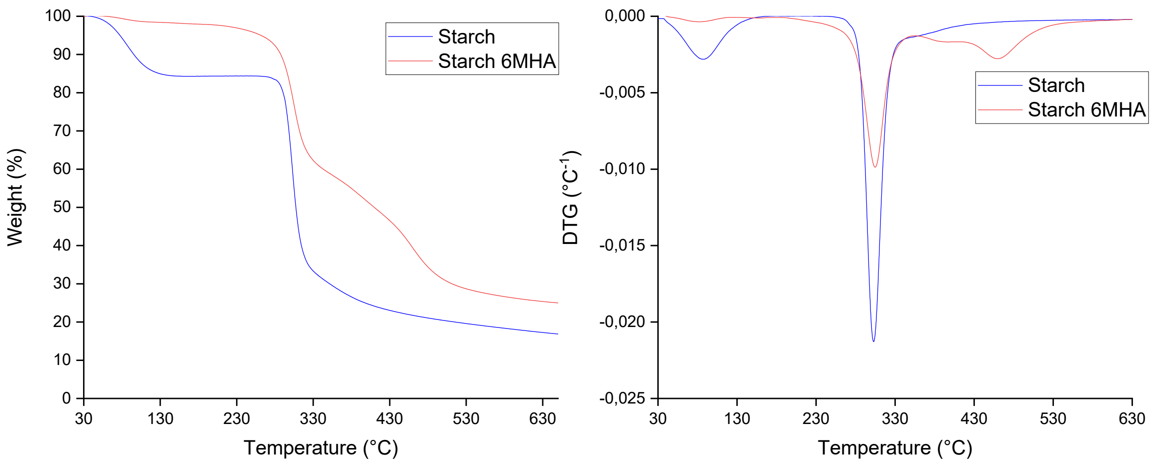

Figure S8. TGA and DTG thermograms of unmodified and modified Starch

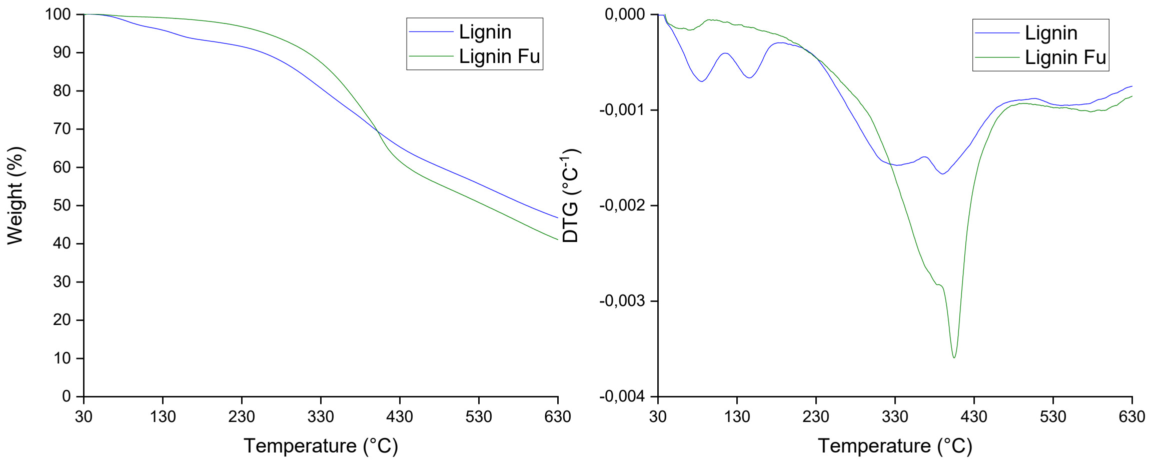

Figure S9. TGA and DTG thermograms of unmodified and modified Lignin

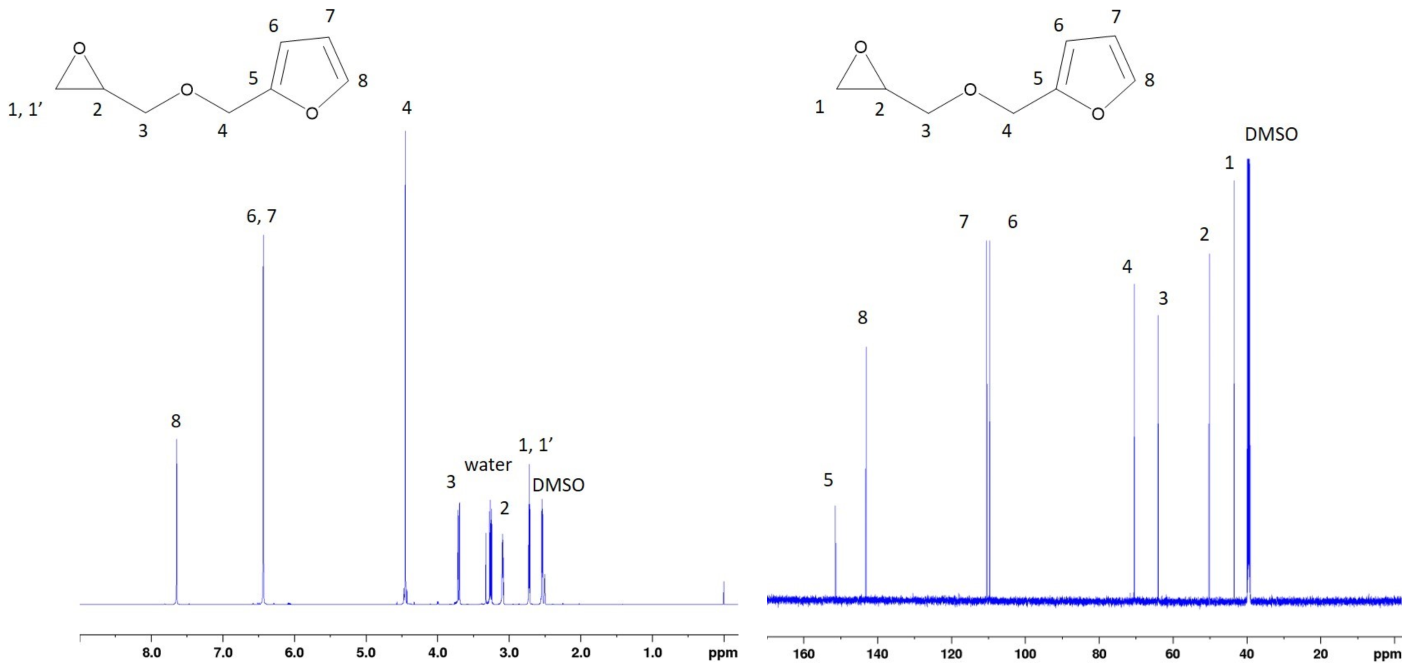

Figure S10. ^{1}H and ^{13}C NMR spectra of Furfuryl glycidyl ether in DMSO-d_6
